# Coarse-Grained Protein Dynamics Studies Using Elastic Network Models

**DOI:** 10.3390/ijms19123899

**Published:** 2018-12-05

**Authors:** Yuichi Togashi, Holger Flechsig

**Affiliations:** 1 Research Center for the Mathematics on Chromatin Live Dynamics (RcMcD), Department of Mathematical and Life Sciences, Graduate School of Science, Hiroshima University, 1-3-1 Kagamiyama, Higashi-Hiroshima, Hiroshima 739-8526, Japan; 2 RIKEN Center for Biosystems Dynamics Research (BDR), 6-2-3 Furuedai, Suita, Osaka 565-0874, Japan; 3 Cybermedia Center, Osaka University, 5-1 Mihogaoka, Ibaraki, Osaka 567-0047, Japan; 4 Nano Life Science Institute (WPI-NanoLSI), Kanazawa University, Kakuma-machi, Kanazawa, Ishikawa 920-1192, Japan; flechsig@staff.kanazawa-u.ac.jp

**Keywords:** elastic network, coarse-grained model, molecular dynamics, normal mode analysis, nonlinearity, protein, molecular machine, allostery

## Abstract

Elastic networks have been used as simple models of proteins to study their slow structural dynamics. They consist of point-like particles connected by linear Hookean springs and hence are convenient for linear normal mode analysis around a given reference structure. Furthermore, dynamic simulations using these models can provide new insights. As the computational cost associated with these models is considerably lower compared to that of all-atom models, they are also convenient for comparative studies between multiple protein structures. In this review, we introduce examples of coarse-grained molecular dynamics studies using elastic network models and their derivatives, focusing on the nonlinear phenomena, and discuss their applicability to large-scale macromolecular assemblies.

## 1. Introduction

Cells are made up of soft materials, and proteins are one of the major components of cells. Besides unfolded and intrinsically disordered proteins, many folded proteins show large-amplitude motions (transitions) between ensembles of conformations. Such conformational motions are often related to their functional cycles such as reactions in enzymes or the translocation of ions in transporters. Hence, to fully understand the mechanism of action of proteins, it is important not only to elucidate their static structures but also to understand their dynamic and mechanical aspects.

A large number of experimental studies have focused on the elucidation of the dynamic and mechanical aspects of various proteins. Nuclear magnetic resonance (NMR) can provide valuable information about protein structures in solution, including their fluctuations. Owing to the recent developments in cryo-electron microscopy (cryo-EM) techniques, realistic structures in solution can now be observed at a high resolution. Atomic force microscopy (AFM) can even visualize the motion of each protein in physiological conditions as a molecular movie [1,2]. It is, however, difficult to pursue both live motion at a high temporal resolution (using a high enough frame rate) and high spatial resolution in a single experiment. This emphasizes the important role of numerical simulations, to integrate and interpret experimental results.

Molecular dynamics (MD) has been used to investigate the motion and mechanical properties of proteins for over 40 years [3]. The accessible time-scale of an all-atom MD for a small protein has been extended from picoseconds to milliseconds [4]. Even complex cytoplasmic environments [5] or whole virus capsids [6] have been modeled and simulated. However, operation cycles of protein machines are slow, typically taking milliseconds to seconds. Even using state-of-the-art supercomputers, all-atom MD simulations beyond the timespan of seconds are currently not realistic. Moreover, due to thermal and hydrodynamic fluctuations at the nano-scale, the operation of proteins is non-deterministic, demanding statistical analyses over a number of functional cycles and simulation trials.

Hence, we still need to reduce the computational cost of molecular dynamics simulations, by coarse-graining and simplification of the model. There is already a huge variety of coarse-grained models for structural dynamics of proteins, at the sub-residue level (e.g., MARTINI [7]), residue level (e.g., many Gō-like models [8,9,10], AICG and variants [11,12]), even coarser or multiscale [13]. Force fields are simplified in different ways in each of these models.

One of the simplest among them is the elastic network model (ENM), in which all the interactions are simplified as springs obeying Hooke’s law. As the interaction potential (force field) depends on a reference structure, ENMs could be seen as a variant of Gō-like models. Despite the radical simplification, ENMs could provide insights into slow conformational motions and relate them to the function of proteins. It is possible to reproduce conformational changes over entire operation cycles. Because of their low computational costs, ENMs are also convenient for comparative studies of many different protein structures. In this review, we briefly explain ENMs and their features, and introduce examples of MD studies using ENMs contributed by us and other groups. We also discuss the improvements in modeling, and further applications beyond single proteins.

## 2. Background

### 2.1. Elastic Network Models

ENMs consist of point-like particles and linear (Hookean) springs connecting the particles. When adopted as a model of a protein, the particles are placed according to a given reference structure of the protein, and then the pattern of connections (springs) is determined by a certain rule. In the simplest form, the network is constructed as follows. To determine the connectivity, the distance between each pair of particles in the reference structure is calculated, and if that value is less than a certain cutoff distance, the pair is connected by a spring; all the springs have the same stiffness constant. The equilibrium length of each spring is set equal to the corresponding distance between the particles in the reference structure so that the reference structure always corresponds to the minimum energy (most stable) state.

The original ENM for proteins by Tirion [14] was an atomic model where each atom is represented by a particle (see also [15]). Coarse-grained ENMs, in which each amino-acid residue is represented by a particle, were then suggested and broadly used [16,17]. To construct a coarse-grained ENM, we usually substitute each α-carbon atom in the reference structure with a particle, and set the cutoff distance ∼10 Å [18]. Below, we use the term ENM for coarse-grained ENMs unless specified. For the mathematical representation, see Appendix A.1.

There are two ways in which ENMs are commonly represented and analyzed: Gaussian network models (GNM) and anisotropic network models (ANM).

If we focus only on fluctuations around the reference structure, and assume that the fluctuations are isotropic, the behavior can be described by the intensity of fluctuations of each particle (regardless of the direction in which the particle fluctuates). Then, the conformational space of the model (with *N* particles) is reduced to be *N*-dimensional (i.e., the directions are lost and only the magnitudes of their displacement are available). The models in this class are called Gaussian network models (GNM), as the isotropic Gaussian form of fluctuations are assumed. They are used for normal mode analysis (NMA; see Section 2.2) to estimate overall fluctuations at each part of the molecule. In the linear regime, the dynamics can be represented by an N×N matrix derived from the connectivity and stiffness. Although the idea of GNM dates back further [19,20], these models were constructed according to an experimentally known structure when applied to proteins.

If we also need the direction in which each particle moves (in 3-dimensional real space), then the conformational space is 3N-dimensional as the (x,y,z)-displacement of each particle is considered. The direction of the force induced by a spring coincides with the direction of the spring, and the intensity of the force is determined by the change in spring length, which is affected by displacement of the particles connected by the springs. Such models are called anisotropic network models (ANM), and are also analyzed by NMA [21,22].

### 2.2. Normal Mode Analysis

To investigate fluctuations or structural transitions in ENMs, NMA is often adopted. In NMA, the motion is linearly approximated around the steady state (i.e., the force on each particle is represented by only the first order of displacement; see Equation (A7) in Appendix A.2), and then decomposed into mutually orthogonal normal modes. This decomposition is mathematically executed by solving the eigenvalue problem of the linearization matrix (i.e., Hessian matrix of the elastic potential energy, scaled by mass or mobility); each eigenvalue shows the frequency (in underdamped cases) or decay rate (in the overdamped limit) of the motion toward the direction of the corresponding eigenvector. Particular focus is on those modes with small eigenvalues, as they represent soft and slow (often large amplitude) motion, typically overdamped in solution [23].

NMA is a general method and has been widely used in molecular mechanics studies along with all-atom models [24,25,26,27,28]. There are several advantages of ENMs when used within the framework of NMA. By definition, the reference structure is always (one of) the state(s) of minimum energy, and thus energy minimization before the analysis is not required. When NMA is applied to GNMs, the N×N connectivity matrix (the Kirchhoff matrix) can be directly used as the linearization matrix.

The dynamics of ANMs in the linear regime is represented by a 3N×3N matrix. In contrast to GNMs, the linearization matrix includes information not only on the connectivity and stiffness but also on the directions of the springs in the reference structure (see Appendix A.2). The force induced by each spring in the linear regime is only toward the direction of the spring in the reference structure, and the intensity is linear to each of x, y, or z-displacement of the particles connected to the spring. Hence, the linearization matrix can be easily obtained (see Equation (A9)).

Since the 1990s, GNM-NMA has been applied to a variety of protein structures, and has successfully reproduced fluctuations of each residue observed in experiments (e.g., the pattern of B-factors obtained from crystallography). Later, ANM-NMA was introduced, and in addition to reproducing the overall intensity of fluctuations, it has been evidenced that the direction of a single (or a few) slow normal modes agreed well with structural changes between two or more known structures at different states (e.g., ATP-bound and ATP-free states in the functional cycle) in protein machines. Owing to its simplicity and extremely low computational costs, these methods became increasingly popular to identify functional conformational motions in protein machines and motors (e.g., with GNM [29,30,31,32] and ANM [30,31,32,33,34]). There are also automated online services [35,36,37,38] and databases [39,40] available for ENM-NMA (see also variants using dihedral angles as independent variables [41,42,43,44]). For details of the methods and further examples, see reviews e.g., [45,46,47,48,49,50,51].

### 2.3. Nonlinearity of ENMs

**Figure A1:**
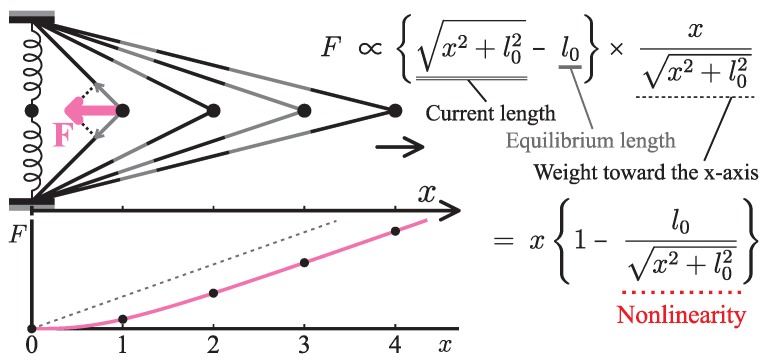
**An illustrative example of the nonlinearity.** Two springs are initially aligned straight without strains; each spring obeys Hooke’s law (equilibrium length: $l_{0})$. If we pull the midpoint perpendicularly to the initial spring direction, the strength *F* of the resultant force F depends nonlinearly on the displacement *x*. Adapted from [62].

Although linear NMA could be successfully applied as discussed above, the dynamics of ENMs is intrinsically nonlinear even though all the springs are linear with respect to their deformations (see Appendix A.1). The nonlinearity arises because the direction of each spring, and consequently the direction of the force induced by the spring, can change through the deformation (see Figure A1 for an illustrative case). Hence, for relatively large deformations, the behavior of ENMs may deviate from the prediction by NMA. The linear response approximation strictly holds only as long as the displacements (changes in the Cartesian coordinates) of network particles from their equilibrium positions are much smaller than the natural lengths of springs. The fact that ENM-NMA for proteins nonetheless describes very well even large-amplitude conformational transitions (which certainly involve large displacements of groups of particles) is remarkable, and may represent a property which is unique to those proteins operating as machines. Still, the validity regime of ENM-NMA in general has to be questioned.

If we consider elastic networks as dynamical systems, their behavior beyond the NMA can be explored. We can perform coarse-grained MD simulations using ENMs, in which each particle is numerically tracked according to Newton’s equation of motion. Obviously, the conformational space of the network has 3N degrees of freedom (the (x,y,z)-coordinates of each particle). In this approach, the full nonlinear elastic dynamics of the network is considered, and effects of nonlinearity can be discussed.

The nonlinear effect was formerly discussed in the context of transitions between structural states, sometimes involving partial unfolding (“proteinquake”) [52]. Attempts were made to connect (interpolate) multiple structures by iteratively applying NMA or introducing a smooth potential surface [53,54,55,56,57]. Also, ENM variants explicitly including nonlinear (higher-order) terms have been developed (e.g., nonlinear network model (NNM) [58]) and used for MD simulations (e.g., [59]).

While the dynamics of compactly folded protein ENMs is intrinsically nonlinear, the question is also to what extent the harmonic restraints between particles in protein ENMs keep their validity. If, e.g., local deformation (strain) of springs is excessive, ENMs are not able to capture possible processes of unfolding. However, if the molecule is sufficiently large, it is possible that its overall deformation is large while local deformation remains very small. In such cases, NMA may break down in the regime where monolithic ENMs (without any interpolation or correction) are still good approximations. Because of the nonlinearity, MD simulations using ENMs may provide additional information beyond the NMA.

### 2.4. ENM-MD Simulations

**Figure 1:**
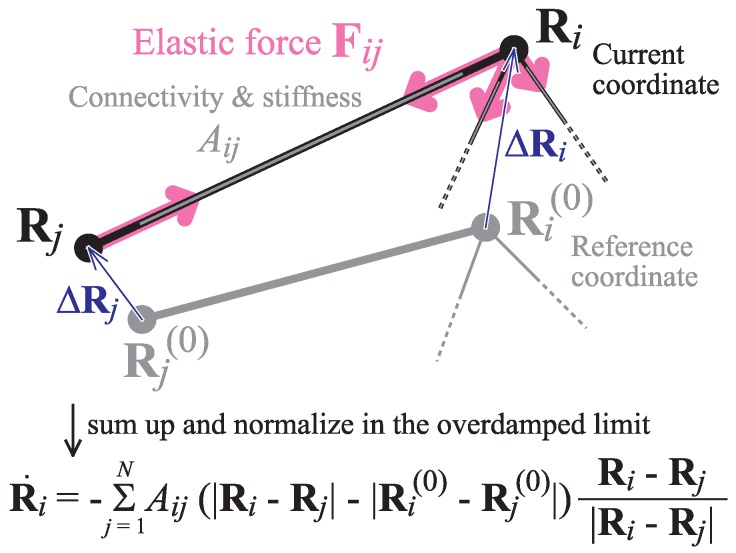
**Schematic diagram of the elastic network model (ENM).** Each spring (i,j) obeys Hooke’s law; i.e., the strength of its elastic force $F_{ij}$ is proportional to its deformation from the equilibrium length, and the force direction coincides with the spring direction. The resultant force $F_{i}$ on particle *i* is the sum of forces $F_{ij}$ exerted by all the springs connected to the particle. In dynamic simulations in the overdamped limit (Section 2.4), the velocity $\dot{R_{i}}$ of particle *i* is directly calculated from $F_{i}$. Adapted from [62].

To consider the effects of nonlinearity, we performed coarse-grained MD simulations using ENMs and compared the trajectories with the results of ANM-NMA [60,61]. Each particle in the network follows Newton’s equation of motion, and the temporal evolution of the network is obtained by numerically integrating the set of coupled equations. When the focus is on slow conformational motion in proteins, inertial effects can be omitted [23], and the equations of motion are considered in the overdamped limit (i.e., the velocity of the particle is proportional to the sum of the forces acting on it; see Figure 1 and Appendix A.1).

**Figure 2:**
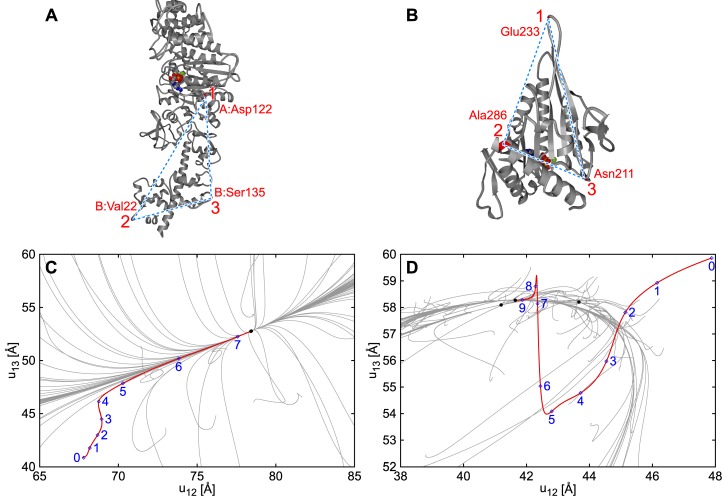
**Relaxation paths of ENMs.** Two molecular motors were compared: (**A**,**C**) Myosin V and (**B**,**D**) KIF1A; (**A**,**B**) Structure of the motors; positions of the markers 1,2,3 are shown. The ATP-bound structure of myosin V and ADP-bound structure of KIF1A were used as the reference structures to construct the ENMs; (**C**,**D**) Trajectories of conformational changes. Horizontal and vertical axes show the distances between markers 1 and 2, and markers 1 and 3, respectively. Gray lines display trajectories starting from 100 different initial deformations, prepared by applying static force toward random direction to each particle. In most cases, these trajectories quickly converge to an energetic valley (represented by a bundle of trajectories in (**C**,**D**)), and slowly return to the reference structure (stable state). This behavior is common for these two motors, and the direction of the valley around the reference structure agrees with the slowest normal mode. Red lines represent transitions between two states of the motors, which start from the nucleotide free state of myosin V (i.e., corresponding to the conformational transition upon ATP binding), and from the ATP-bound state of KIF1A (i.e., corresponding to the transition from the ATP-bound state to the ADP-bound state); label 0 shows the initial condition and the relaxation proceeded 1, 2, …. In myosin V, the trajectory was similar to those from random deformations, and the normal mode approximation holds well except for initial changes. In KIF1A, in contrast, the relaxation progressed with multiple steps, and the trajectory converged to the slowest normal mode direction only at the final stage of relaxation (<1 Å of distance change). Reproduced from [61] with modification.

Figure 2 shows examples of two different classes of molecular motors: myosin V and kinesin KIF1A [61]. Starting from random deformations, in most cases, the trajectories quickly converge to an energetic valley, and slowly go back along the valley to the reference conformation. If we start from the conformation in another state, we can simulate the transition between the states (e.g., if the ENM is constructed according to the ligand-bound state and the simulation starts from the ligand-free conformation, then the simulation mimics the motion after ligand binding). Also in this case, the trajectories go along a well-defined pathway and are robust against fluctuations. In both cases, the behavior at the final stage agrees with the slowest mode in the NMA.

In some molecules however, we found that the region where NMA holds (i.e., the behavior is largely linear) is extremely small. In the case of KIF1A, a sequence of motion caused by steric hindrance was observed between two structural states; NMA is applicable only to the final stage of the motion. Still, the deformation of each spring was small compared to the length in the reference conformation, which supported the validity of the ENM.

## 3. Applications

In this way, ENMs are used for coarse-grained MD simulations, which provides information on the structural dynamics beyond NMA. Although the inherent nonlinearity may show physically interesting behavior apart from biology (e.g., glass-like, nonequilibrium behavior was observed in ENM-MD with thermal noise [63]), here we introduce examples of ENM-MD applied to more physiologically important problems.

### 3.1. Probing Dynamical Basis of Allostery

In these simulations using ENMs, in the same way as other models, we can apply any external forces in MD simulations (so-called steered MD simulations [64]), and also measure forces acting on any particle or spring (cf. in the linear regime, the response could be considered by ANM-NMA [65,66]). It is analogous to force probing experiments using AFM. Force-extension curves can be obtained in a similar way to AFM experiments. We can apply force (or deformation) at any part of the molecule and detect the resulting deformation or strain generated in any other parts. For example, mechanical properties of transcription activator-like effector (TALE) proteins were considered by stretching simulations [67].

As a direct application of this method, effects of external forces on molecular machines were considered. In myosin motors, it was experimentally reported that the affinity to actin filaments is modulated by the force applied to the tail (strain sensor) [68]. The actin binding domain is so far away from the tail (∼10 nm) that mechanical communication between these domains is likely to exist, however the mechanism was not known. An external force was applied to the tail to mimic the experiments, and structural changes in the other parts were observed [69]. It was observed that the actin-binding interface was affected by the force. Moreover, as the computational cost is very low, we could even try applying a force to every particle (residue) in the molecule, which could systematically show the connections between the tail, actin-binding interface, and the ATP binding pocket.

**Figure 3:**
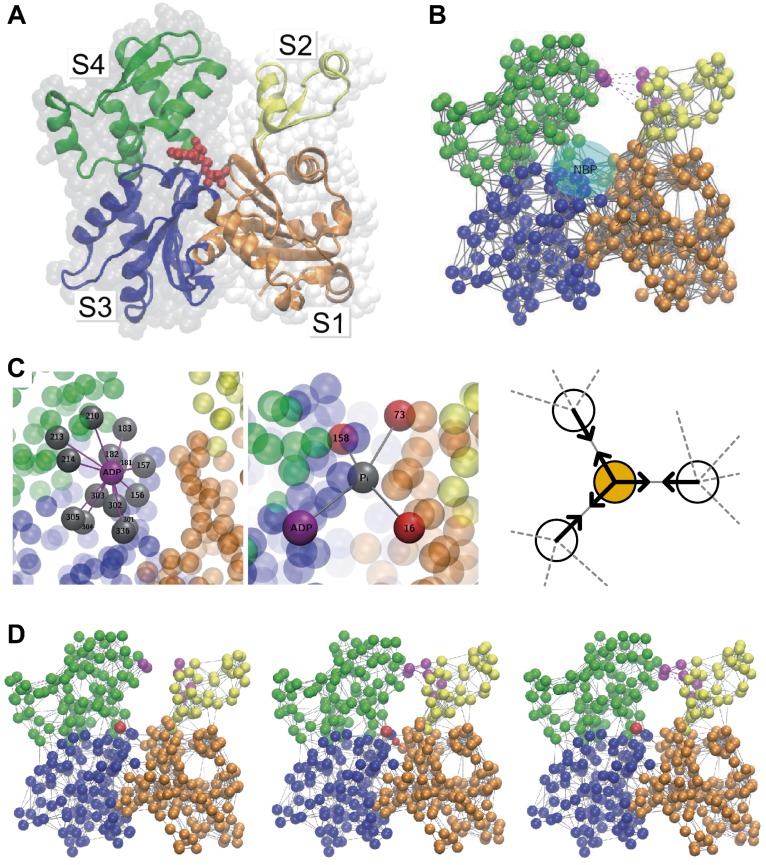
**Ligand-induced conformational changes simulated by ENMs.** (**A**) structure of G-actin and (**B**) its ENM representation; (**C**) In the nucleotide binding pocket (NBP), two additional particles (ADP and P${}_{i}$) were introduced to mimic ligand binding. The natural lengths of springs connected to P${}_{i}$ (solid lines in the schematic) was set shorter than the distances in the reference structure, so that they introduce attractive forces (arrows), mimicking the shrinkage of the NBP upon ATP binding; (**D**) The motion introduced by the ligand, together with additional interactions (breakable links shown in purple lines), suggested ATP-induced transition to the closed conformation (**left** to **center**), which may explain the acceleration of actin polymerization by ATP. A metastable closed state after ATP hydrolysis and P${}_{i}$ release (**right**) was also observed. Reproduced from [71] with modification.

This method is also applicable to study the effects of ligands, if the ligand binding can be mimicked by additional particles and springs. This approach was first developed to study the full operation cycles of hepatitis C virus helicase motor [70], which is reviewed in Section 3.2. It was also applied to actin, to study the mechanism of ATP-induced conformational changes and their effects on polymerization [71] (see Figure 3). In this work, ATP was modeled as two additional particles (ADP and P${}_{i}$ in Figure 3C) connected by a spring. In a slightly different way, ATP-induced conformational changes of ABC transporters were modeled and discussed [72]. As ENM-MD is effective at simulating long-time behavior, it could be used together with short all-atom MD simulations for studying structural details. A combination of targeted all-atom MD simulations and targeted ENM-MD simulations including the effect of ATP binding was adopted to investigate the inter-subunit coupling of F${}_{1}$-ATPase [73].

Furthermore, this method can be applied theoretically to any kind of protein structure. The low computational cost makes it feasible to exhaustively analyze communication patterns inside the molecule for a variety of protein structures deposited in the Protein Data Bank, which may enable us to systematically study the structural basis of allosteric regulation [74].

### 3.2. Tracing Entire Motor Cycles

Protein machines couple chemical events of nucleotide binding and hydrolysis to cyclic conformational motions, performed to implement a particular function. In motor proteins, such motions are used to exert forces on other proteins (such as filaments or nucleic acid strands) and produce mechanical work. Since cyclic internal motions are typically slow (with timescales ranging from milliseconds to seconds), all-atom MD simulations are not applicable despite the use of supercomputers. We used elastic networks considered as dynamical systems (beyond the NMA) to investigate the full internal operation of a motor protein.

**Figure 4:**
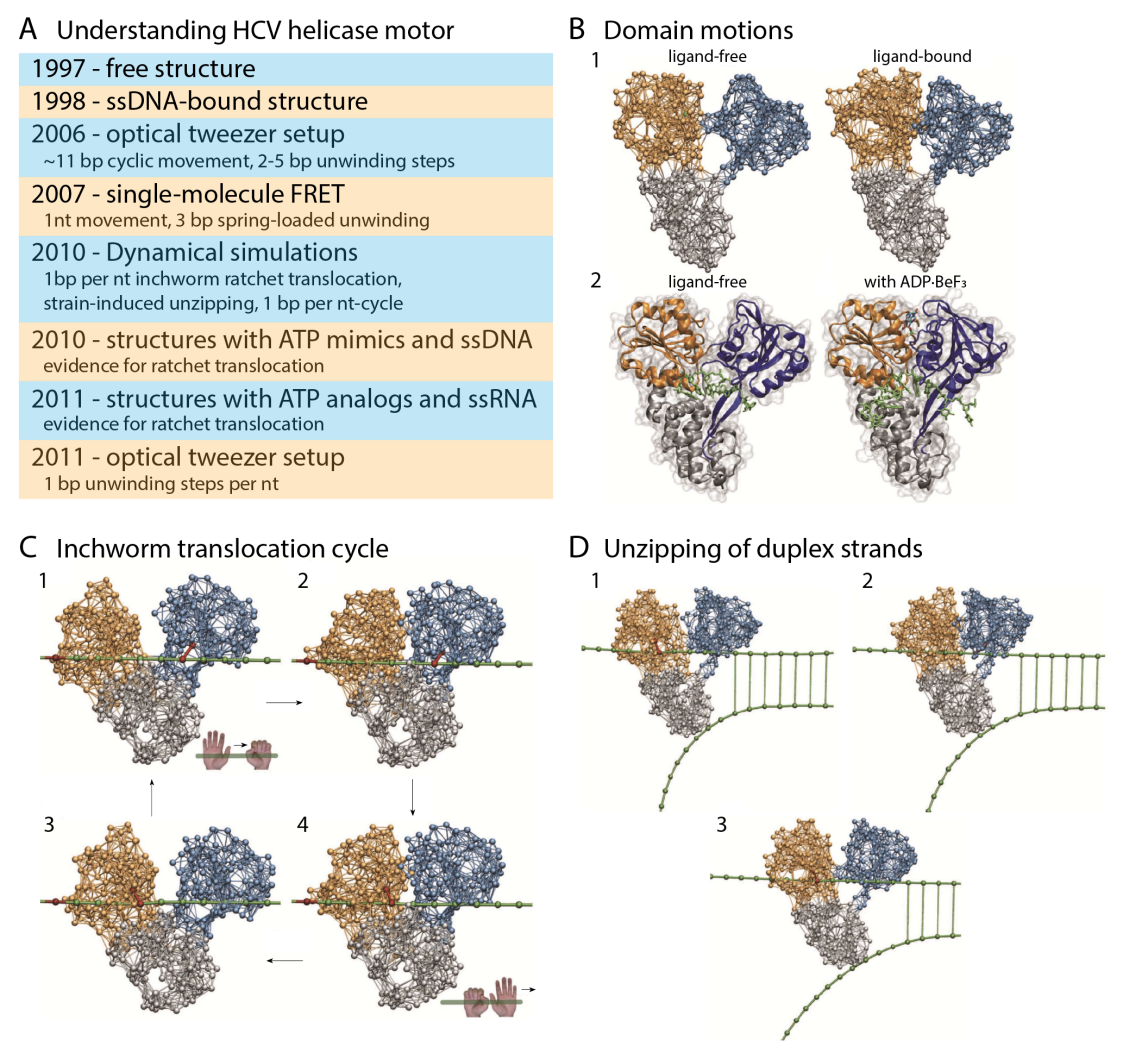
**Entire operation cycles from ENM.** (**A**) Table showing the main experimental efforts and observed/proposed mechanisms for the operation of the hepatitis C virus (HCV) helicase molecular motor. The references for this table are [70,77,78,79,80,81,82,83]; (**B**–**D**) Summary of results of structurally resolved dynamical simulation for HCV helicase based on ENM modeling of this motor; (**B**) Structures obtained from our simulations are compared with crystal structures from [81]; (**C**) Ratcheting inchworm translocation observed in the simulations is shown as snapshots from a single cycle. Employing the alternating hand-on hand-off mechanism of grip control on the nucleic-acid strand, the motor domains can translate their nucleotide-related internal opening and closing motion into their transport by 1 base-pair (bp) per consumed nucleotide; In (**D**), coupling of inchworm translocation to mechanical separation of duplex strands is shown as snapshots [70,75].

A modeling scheme which allowed us to follow ATP-induced operation cycles in molecular machines was developed and implemented to trace the operation cycles of hepatitis C virus (HCV) helicase molecular motor (see Figure 4). Very few structures of this motor were available at that time, and although single molecule experiments were performed, there was no consensus about the operation mechanism employed by this motor. Our elastic-network based simulations revealed the functional conformational motions which are used by this motor to translocate along single nucleic-acid strands and, at the same time, unzip duplex regions [70,75,76]. Based on these simulations we could identify the mechanism by which HCV helicase operates (see Figure 4). Our simulations could confirm the ratcheting inchworm mechanism of helicase translocation, which was previously proposed for this motor based on single-molecule experiments. The mechanism of strand separation observed from our structurally resolved simulations further supported the previously hypothesized spring-loaded unwinding mechanism.

After our study was reported, a variety of novel crystal structures which captured the HCV helicase motor in complex with nucleic acid substrates and together with ATP-analogs became available [81,82]. These experimental results provided further evidence for the ratcheting translocation. Furthermore, results of higher-resolution optical tweezer experiments reporting single base-pair steps of unwinding (as also seen in our coarse-grained simulations) were published [83]. Molecular simulations performed at the atomistic level provided further insights into details of the inchworm transport mechanism used by HCV helicase [84,85].

## 4. Further Improvement of Models and Simulation Methods

In conventional ENMs, the stiffness constant is typically the same for all springs, sometimes larger for the backbone, but not depending on the residue type or distance. In other words, all the chemical properties are omitted, unless implicitly embedded in the reference structure. It is a radical simplification, as the interactions between the sidechains depend on the type of residues; e.g., electrostatic interactions may be significant between charged residues.

Hence, efforts have been made to take into account such differences in residues in ENMs. In early days, the cutoff distance and spring constants were typically determined to maximize the correlation between the predicted intensity of fluctuations and the B-factor observed in crystallography for each residue [86]. However, this strategy relied on the crystallographic B-factor, which may not reflect the actual fluctuations in solution at room temperature. Conformational changes between two known structures were also used for the evaluation, particularly for ANMs [87]. Information on chemical bonds (e.g., hydrogen bonds between the residues) was also used to determine the parameters. To solve the cutoff dependency, a parameter-free version of ENM was also proposed [88].

Later, Dehouck and Mikhailov took another approach [89]. They extracted information on structural fluctuations from conformational ensembles obtained by NMR. Using this information, they determined the effective stiffness value, as a function of the pair of residue-types and the distance in the reference structure. As this method depends on solution NMR experiments, effects of thermal and hydrodynamic fluctuations and also hydrophobic interactions in solvent are effectively included. Besides the heterogeneous stiffness constants, a standard ENM description is employed. Hence, we can enjoy the benefits without much additional computational cost.

The construction of a protein ENM depends on the reference structure. However, although NMR and cryo-EM techniques as well as conventional crystallography have drastically evolved, high-resolution structures are not always available. ENMs could be successfully constructed from low-resolution structures [90,91], and then used for structure refinement (e.g., [92,93], followed by many others). It was also suggested that the overall shape is crucial for the determination of slow molecular motion [94]. Even further coarse-graining was possible as long as the shape was conserved [95,96,97] (although evaluation in these early works depended on NMA, it was suggested that slow normal modes are robust against amino-acid sequence variations [98]). This property may be beneficial when modeling a huge molecular complex such as chromatin (see Section 5).

Other than ENMs, there are many models similar to or related to ENMs. A major drawback of ENMs is that the structure cannot unfold, as the interactions are approximated by Hookean springs. When applied to multidomain proteins that can partially unfold, or include intrinsically disordered regions, breakable bonds represented by the Lennard-Jones potential are often introduced to allow unfolding. Such variants have also been used for ENM-MD simulations (e.g., [99]). This idea dates back to the 1990s (e.g., [100]), and is also related to off-lattice Gō-like models [8,9] (again, ENMs including these variants could be seen as a kind of Gō-like models, as the force field depends on a reference structure; for a perspective on MD studies using such models, see reviews e.g., [13,101]). In the examples above, to reproduce transitions between two structures, we used one structure as the reference and the other as the initial condition. This is of course a crude simplification, and smoother morphing methods have also been introduced [53,54,55,56,57]. Multiscale hybrid methods (e.g., all-atom MD and ANM-NMA [102]) have been developed, and combination with ENM-MD is possible.

Finally, ENMs can be combined with models of other kinds of molecules. For example, in [103], an ENM was coupled with solvent molecules represented by multiparticle collision dynamics (MPC), to consider effects of hydrodynamic fluctuations. It was shown that the conformational changes can be ordered and accelerated by hydrodynamic fluctuations (i.e., collective motion of solvent molecules). Combined with a morphing method, it was applied to adenylate kinase, which shows the relatively large open-close motion of multiple domains [104]. Again, the hydrodynamic effect alters the motion in the reaction cycles. Swimming-like behavior due to the asymmetric cyclic motion was also observed. Effects of crowding (obstacles) could be also incorporated [105]. Using MPC dynamics, substrate and/or product molecules as well as the solvent could be explicitly included into the simulation of an enzyme; e.g., enzyme kinetics of phosphoglycerate kinase was simulated [106], and reactivity of multiple active sites in 4-oxalocrotonate tautomerase was investigated [107]. External interactions such as electrostatic and van der Waals forces can also be taken into account, to study the interactions between molecules (e.g., actin and myosin [108]).

## 5. Discussion

To summarize, ENMs are of wide use not only for analysis of structural fluctuations using NMA but also for reproducing dynamic processes using molecular dynamics, and even entire machine operation cycles.

The dynamic properties of proteins were compared with those of random or designed artificial elastic networks [60]. It was later reported that ENMs constructed for fractal polymer globules also showed relaxation dynamics similar to that of protein ENMs [109]. Furthermore, designed elastic network structures that reproduce “protein-like” features can be considered and compared with ENMs of real proteins. For example, machine-like motions induced by ligand binding were demonstrated [60], and models of linear motors were constructed [110,111]. ENMs mimicking allosteric effects could be constructed through evolutionary optimization of the structure [112]. Although it is beyond the scope of this review, we hope this direction of study will help to elucidate the design principles of protein structures, independent of the history of evolution. For a discussion of those aspects, we refer the reader to [113].

Recently, ENMs have also been used for biomolecules other than proteins. Complexes of proteins and nucleic acids such as ribosomes [114] and nucleosomes [115] were modeled as ENMs, and their dynamics were analyzed by NMA. Structures of free DNA double-strands were predicted from their sequences and modeled as ANMs (at different coarse-graining levels where each particle represents an atom to several bases), and their structural fluctuations were analyzed by NMA [116,117]. Furthermore, GNM was applied to chromatin structure (DNA-protein complex in the cell nucleus) [118]. Each particle corresponds to kilobases of DNA, which is much larger than an amino acid residue in proteins. Despite the difference in resolution, the mobility of genomic loci was successfully predicted by NMA in a similar way as for proteins. In this particular case, GNM has an advantage that it could be constructed from only the neighbor matrix obtained by Hi-C experiments; nonetheless, with 3D structure reconstruction, ANM-type models can also be constructed from the experimental data, which can also be readily used for dynamic simulations and will provide more information on the structural fluctuations.

Using all-atom MD simulations is still challenging and needs specialized hardware to exceed milliseconds even for a small protein [4,119]. In high-performance computing (HPC) using massively parallel supercomputers, it is generally much harder to utilize the parallelism for solving the same size of problem faster (strong scaling) than solving a larger problem within the same time (weak scaling). Assuming the same computational effort and lapse-time, the former represents simulations of long-time behavior of a small protein, while the latter corresponds to simulations of a huge complex for a short period. Considering the current trend of HPC depending on huge parallelism, such low-cost methods using ENMs and other simplified models will remain useful, serving as a realistic and convenient choice in the future.

## Appendix A. Mathematical Representation of the Models

### Appendix A.1. Elastic Network Models

As introduced in Section 2.1, ENMs consist of point-like particles connected by springs. To construct an ENM for a protein, a structural data set (typically chosen from the Protein Data Bank) is used as the reference. In the simplest form, coarse-grained ENMs are constructed and mathematically represented as follows (see also Figure 1).

The coordinate of each α-carbon atom in the reference structure is used as the equilibrium position $R_{i}^{\left(0\right)}$ of the corresponding network particle. For each pair of particles, if the distance $d_{ij}^{\left(0\right)}\;=\;\left|R_{i}^{\left(0\right)}-R_{j}^{\left(0\right)}\right|$ in the reference structure is less than the cutoff distance $l_{0}$, the pair is connected by a spring; the equilibrium length of the spring is equal to $d_{ij}^{\left(0\right)}$, i.e., springs are not strained in the reference conformation. All the springs have the same stiffness constant κ.

Each spring obeys Hooke’s law. The force direction coincides with the spring direction (without torsion). Then, the spring that connects particles *i* and *j* exerts force

(A1) $$F_{ij}=-\kappa \left(d_{ij}-d_{ij}^{\left(0\right)}\right)\frac{R_{i}-R_{j}}{d_{ij}}$$

on particle *i*. Here, $R_{i}$ is the current coordinate of particle *i*, and $d_{ij}=\left|R_{i}-R_{j}\right|$ is the actual distance between particles *i* and *j*. Thus, the resultant force acting on particle *i* is

(A2) $$F_{i}=-\kappa \sum_{j=1}^{N}A_{ij}\left(d_{ij}-d_{ij}^{\left(0\right)}\right)\frac{R_{i}-R_{j}}{d_{ij}},$$

where *N* is the number of particles in the network, and the adjacency matrix $A_{ij}=1$ if $d_{ij}^{\left(0\right)}<l_{0}$ and $A_{ij}=0$ otherwise. It should be noted that, although the forces (A2) are linear in terms of changes in the spring lengths, they are still nonlinear because distance $d_{ij}$ has nonlinear dependence on the spatial coordinates, i.e., $d_{ij}=\sqrt{{\left(x_{i}-x_{j}\right)}^{2}+{\left(y_{i}-y_{j}\right)}^{2}+{\left(z_{i}-z_{j}\right)}^{2}}$. The total elastic energy of the network is

(A3) $$U=\frac{\kappa }{2}\sum_{i<j}A_{ij}{\left(d_{ij}-d_{ij}^{\left(0\right)}\right)}^{2}.$$

In ENM-MD simulations (Section 2.4), Newton’s equations of motion with the forces represented by Equation (A2) are numerically solved. When we consider slow conformational dynamics of proteins in the solvent, the motion is assumed to be overdamped [23]. We also ignore hydrodynamic effects (cf. combination with MPC solvent [103]). Then, in the overdamped limit (i.e., the velocity of each particle is proportional to the total force acting on it), the motion of particle *i* (at time *t*) is governed by

(A4) $$\frac{dR_{i}}{dt}=\Gamma_{i}F_{i},$$

where $\Gamma_{i}$ is the mobility. Here, we assume the same mobility for all the particles, i.e., $\Gamma_{i}=\Gamma$. With rescaling of time (measured in units of ${\left(\Gamma \kappa \right)}^{-1}$), the dynamics are explicitly represented by

(A5) $$\frac{dR_{i}}{dt}=-\sum_{j=1}^{N}A_{ij}\left(\left|R_{i}-R_{j}\right|-\left|R_{i}^{\left(0\right)}-R_{j}^{\left(0\right)}\right|\right)\frac{R_{i}-R_{j}}{\left|R_{i}-R_{j}\right|}.$$

Figure 2 shows relaxation dynamics simulated in this way [61]. Random initial deformations (for gray lines) were prepared by simulations under additional static forces, starting from the reference conformation. Specifically, random static forces $f_{i}$ acting on particle *i* were independently generated with the constraint such that $\sum_{i}{\left|f_{i}\right|}^{2}$ is constant (designated force intensity), and then

(A6) $$\frac{dR_{i}}{dt}=f_{i}-\sum_{j=1}^{N}A_{ij}\left(\left|R_{i}-R_{j}\right|-\left|R_{i}^{\left(0\right)}-R_{j}^{\left(0\right)}\right|\right)\frac{R_{i}-R_{j}}{\left|R_{i}-R_{j}\right|}.$$

was used instead of Equation (A5). After a certain length of simulation, the final conformation was in turn used as the initial condition for the relaxation simulation.

### Appendix A.2. Normal Mode Analysis

NMA is often adopted for ENMs (Section 2.2). In NMA, the motion is linearly approximated around the steady state, and then decomposed into normal modes. The forces are linearly approximated in terms of displacements, and represented by using a linearization matrix. For conservative forces, it is equal to the Hessian matrix of the potential energy. Each eigenvalue of the matrix shows the frequency (in underdamped cases) or decay rate (in the overdamped limit) of the motion toward the direction of the corresponding eigenvector.

When NMA is applied to GNMs with uniform stiffness and mass (mobility), we can simply use the Kirchhoff matrix (connectivity matrix). For ANMs, we provide a brief explanation below.

If deviation $\Delta R_{i}=R_{i}-R_{i}^{\left(0\right)}$ from the reference conformation is sufficiently small for all the particles, Equation (A5) can be linearized as

(A7) $$\frac{d}{dt}\Delta R_{i}=-\sum_{j=1}^{N}A_{ij}\left[u_{ij}^{\left(0\right)}\cdot \left(\Delta R_{i}-\Delta R_{j}\right)\right]u_{ij}^{\left(0\right)},$$

where $u_{ij}^{\left(0\right)}=\left(R_{i}^{\left(0\right)}-R_{j}^{\left(0\right)}\right)/d_{ij}^{\left(0\right)}$; i.e., the change in spring length can be linearly approximated as the inner product of the unit vector $u_{ij}^{\left(0\right)}$ toward the spring direction in the reference conformation and the relative displacement vector $\left(\Delta R_{i}-\Delta R_{j}\right)$ (see Figure 1). It is worthwhile to compare the linearized forces in Equation (A7) which have static directions $u_{ij}^{\left(0\right)}$, with the exact forces in Equation (A5) which act along the actual orientations of the springs.

The set of linearized Equation (A7) should be a good approximation as long as the deviations (changes in the Cartesian coordinates) $\Delta R_{i}$ are much smaller than the equilibrium lengths $d_{ij}^{\left(0\right)}$ of springs between particles. Therefore, by comparing the coordinate changes with the typical equilibrium lengths, we can formulate an estimate for the validity of the approximation. In a typical protein ENM with cutoff distance 10 Å, we can assume (as a rough estimate) a value of 5 Å for the average equilibrium length of springs. Then, the linear description should hold for coordinate changes much smaller than that value, i.e., they should not be larger than, e.g., 10% of it (0.5 Å).

Equation (A7) is represented in the matrix form

(A8) $$\frac{d}{dt}\Delta R_{i}=-\sum_{j=1}^{N}\Lambda_{ij}\Delta R_{j}.$$

Λ is a 3N×3N matrix:

(A9) $$\Lambda_{i\sigma ,j\eta }=A_{ij}u_{ij,\sigma }^{\left(0\right)}u_{ij,\eta }^{\left(0\right)}\;\left(\mathrm{for}\;i\neq j\right),\;\Lambda_{i\sigma ,i\eta }=-\sum_{j}A_{ij}u_{ij,\sigma }^{\left(0\right)}u_{ij,\eta }^{\left(0\right)};$$

where σ,η∈{x,y,z}. Note that this result is equivalent to the Hessian matrix of the potential *U* in Equation (A3) with rescaling.

The general solution of Equation (A8) is given as the superposition of 3N−6 exponentially decaying normal modes, i.e.,

(A10) $$\Delta R_{i}\left(t\right)=\sum_{\alpha =1}^{3N-6}k_{\alpha }e^{-\lambda_{\alpha }t}e_{i}^{\left(\alpha \right)},$$

where $\lambda_{\alpha }$ are the eigenvalues, and $e_{i}^{\left(\alpha \right)}$ are the corresponding eigenvectors, of matrix Λ. Each eigenvalue $\lambda_{\alpha }$ represents the decay rate of the normal mode (cf. in the underdamped case, $\lambda_{\alpha }$ represents the vibrational frequency).

Matrix Λ has 3N eigenvalues, however 6 of them are always zero, corresponding to the global translation and rotation. Note that instead of Cartesian coordinates, internal coordinates such as dihedral angles can be chosen as variables [42,44,120,121], which dates back to early works e.g., [24]. At the cost of simplicity of the potential function and its Hessian, the degree of freedom could be drastically reduced; that is beneficial for large models for which the computational cost for the eigenvalue problem (matrix diagonalization) is huge.
